# Diversity and pathogenicity of *Fusarium* species associated with *Fusarium* head blight in wheat and maize cropping systems in Sichuan Province

**DOI:** 10.1038/s41598-024-83402-7

**Published:** 2025-02-18

**Authors:** Xiaofang Sun, Rui Yang, Huimin Tang, Miaomiao Ma, Huabao Chen, Xiaoli Chang, Min Zhang, Guoshu Gong

**Affiliations:** 1 Plant Protection Department, College of Agronomy, Sichuan Agricultural University, Chengdu, 611130 China; 2 Industrial Crops Research Institute, Sichuan Academy of Agricultural Sciences, Chengdu, 610300 China

**Keywords:** *Fusarium* head blight, Wheat‒maize cropping system, Wheat straw, Maize stubble, Population composition, Pathogenicity assays, Fungi, Pathogens

## Abstract

*Fusarium* head blight (FHB) is a severe disease worldwide that leads to substantial economic losses. Wheat‒maize cropping is the dominant system in Sichuan Province, China. However, FHB has become increasingly severe in this system, and *Fusarium* rot disease is also becoming a severe threat to maize. To understand the composition and pathogenicity of the *Fusarium* species associated with FHB, samples of typical symptomatic wheat spikes were collected from wheat‒maize cropping fields in 16 administrative districts of Sichuan Province, and *Fusarium* perithecia were obtained from both wheat straw and maize stubble. Based on morphological and molecular identification, 175 isolates from symptomatic wheat spikes were identified as five *Fusarium* species: *F. asiaticum*, *F. avenaceum*, *F. graminearum*, *F. meridionale*, and *F. proliferatum*. Among them, *F. asiaticum* and *F. graminearum* were the dominant pathogenic species, with isolation frequencies of 75.43% and 20.57%, respectively. Additionally, 136 single-ascospore isolates from wheat straw or maize stubble were identified as *F. asiaticum*, *F. equiseti*, *F. graminearum*, *F. meridionale*, *F. proliferatum*, and *F. temperatum*. Pathogenicity assays revealed that the *Fusarium* strains from all sources could successfully infect wheat and maize. *F. graminearum* exhibited a high degree of pathogenicity towards both crops under investigation, while *F. asiaticum* demonstrated significantly greater pathogenicity towards wheat than maize. This work will help understand the cyclic infection caused by *Fusarium* species in wheat‒maize cropping systems and provide valuable data for the effectively controlling *Fusarium* rot disease in both wheat and maize.

**Supplementary Information:**

The online version contains supplementary material available at 10.1038/s41598-024-83402-7.

## Introduction

*Fusarium* head blight (FHB), also known as wheat scab, is a destructive disease of wheat worldwide. *Fusarium* species can not only infect kernels, thus reducing the yield of wheat and other small-grain cereals, but also produce mycotoxins that are harmful to humans and animals^1,2^. FHB was first reported in 1936 in China, and the Yangtze River valleys were known as the most severe FHB epidemic areas for an extended period^3–5^. The disease has spread northward since the last decade and is now in the Huanghuai region, which is currently the primary wheat production area of China^6^. Over the past decade, the annual average area affected by *Fusarium* head blight has exceeded approximately 4 million hectares, especially in 2012, when there was a severe outbreak^7,8^.

The causal agents of FHB are multiple species of *Fusarium*, such as *F. acuminatum*, *F. asiaticum*, *F. avenaceum*, *F. culmorum*, *F. equiseti*, *F. graminearum*, *F. poae*, *F. verticillioides*, and *F. tricinctum*^9–11^. *F. graminearum* is the primary causal agent of FHB in North America and Australia^12,13^. However, except for *F. graminearum*, *F. meridionale* and *F. poae* are significant causative agents of FHB in southern Brazil^14,15^. In Europe, the *F. graminearum* species complex (FGSC) has become increasingly prevalent in wheat^16–18^. *F. asiaticum* and *F. graminearum* are the predominant pathogens of FHB in Asia^19–21^. In China, at least 15 Fusarium species have been reported to cause FHB, among which *F. graminearum* and *F. asiaticum* are dominant^1,5,7,19,22^. Usually, *F. graminearum* is the most prevalent species in the cooler region of northeastern China, whereas *F. asiaticum* is widely distributed in the warmer area of southern China. Therefore, climate and geographical location are likely pivotal factors in the prevalence of FHB epidemics^23^.

Crop rotation is a common agricultural practice; effective crop rotation practices improve soil fertility in low-productivity soils^24^. Moreover, strategic crop rotation reduces nitrogen fertilizer use and environmental losses, boosts grain yield, and supports sustainable agriculture and environmental health^25,26^. A wheat-maize rotation system can reduce weed density and biomass, reducing the dosage of herbicides and other chemical pesticides while also increasing grain yield^27,28^. During the last decade, the leftover straw from crop rotation has typically been treated by returning it to fields. As an important measure for improving soil structure and increasing soil fertility, this practice has been widely promoted and adopted in China^24,29^. However, this has led to a significant increase in the number of pathogen sources in the field, which increases the risk of disease prevalence and disaster^30^. Specifically, in wheat‒maize cropping rotation systems, the incidence of *Fusarium* maize stalk rot (MSR) and ear rot, as well as FHB in wheat, has increased over time^31,32^. Many studies indicate that continuous wheat cropping or a cropping system of wheat following maize significantly increases the incidence and severity of FHB^12,33–35^. Besides, crop residues (e.g., wheat straw, maize stubble and other host plants) can provide natural hotbeds for *Fusarium* species, increasing FHB risk. These residues may be necessary for genetic exchange between cereal and noncereal populations^31,36,37^.

Wheat‒rice and wheat‒maize cropping systems have long coexisted in Sichuan Province, China. However, in the past decade, wheat‒maize cropping systems have gradually become more popular because of the promotion of straw return and no-tillage practices. Currently, research on the host selection of *Fusarium* in cyclic infection between wheat and maize is somewhat limited. In this planting system, crop residues carrying *Fusarium* propagules are considered inoculum sources for FHB and MSR. However, the relations between FHB and crop residues remain unknown, and it is unclear whether all *Fusarium* species are pathogenic to subsequent crops. Therefore, the objectives of this study were (1) to define the population composition of *Fusarium* species in the wheat‒maize cropping system and (2) to determine the aggressiveness of *Fusarium* isolates on both wheat straw and maize stubble by cross-infection.

## Results

### Identification of *Fusarium* species in a wheat‒maize cropping system

**Fig. 1:**
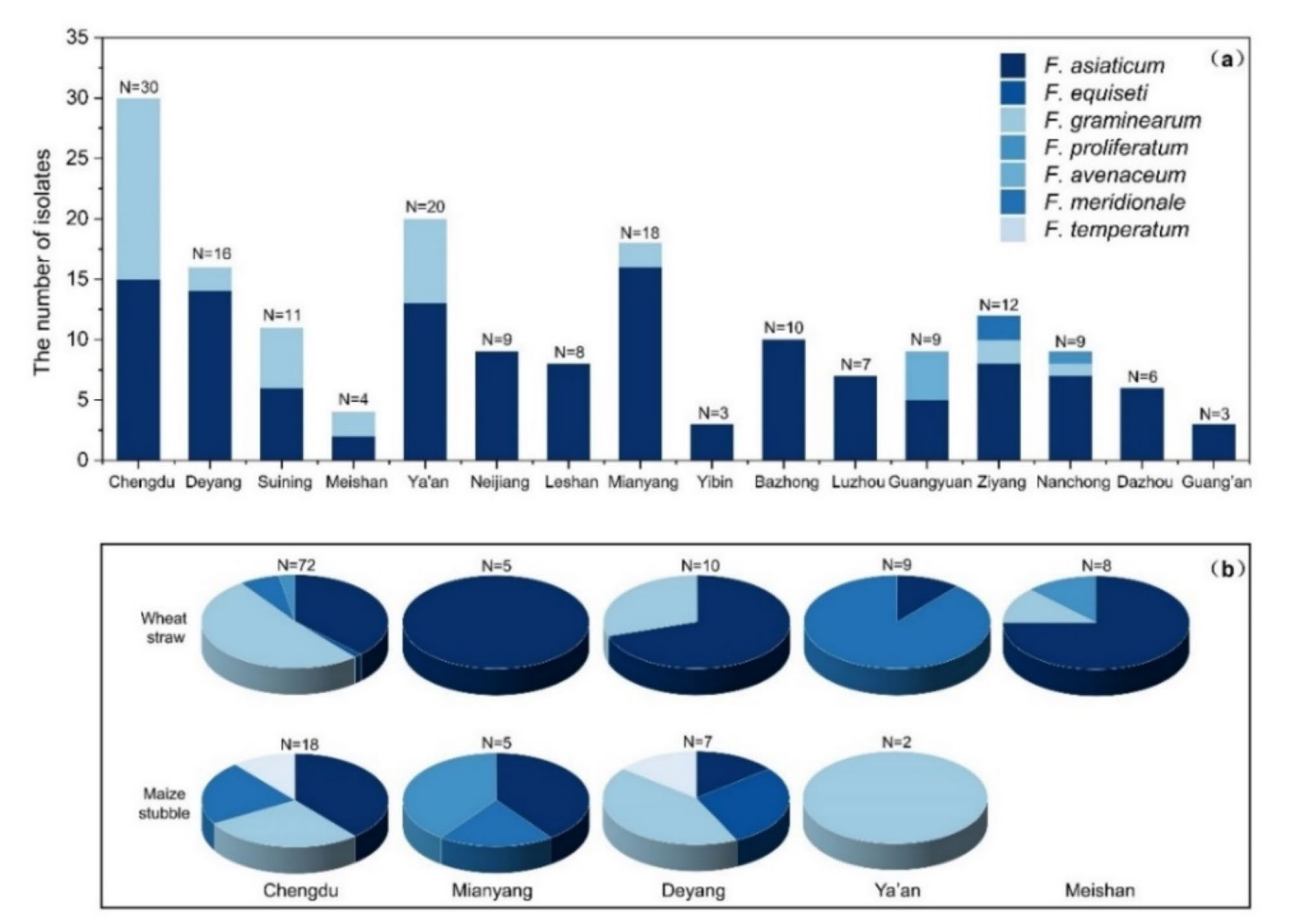
Geographical distribution of the isolated *Fusarium* species according to different sources in Sichuan Province, China. (**a**) *Fusarium* species isolated from *Fusarium* head blight. (**b**) *Fusarium* species isolated from wheat straw and maize stubble. N: number of isolates collected from each region.

A total of 311 single-spore isolates identified morphologically as *Fusarium* spp. were obtained (Fig. 1). The details of all single-spore isolates are listed in supplementary Table S1.

**Fig. 2:**
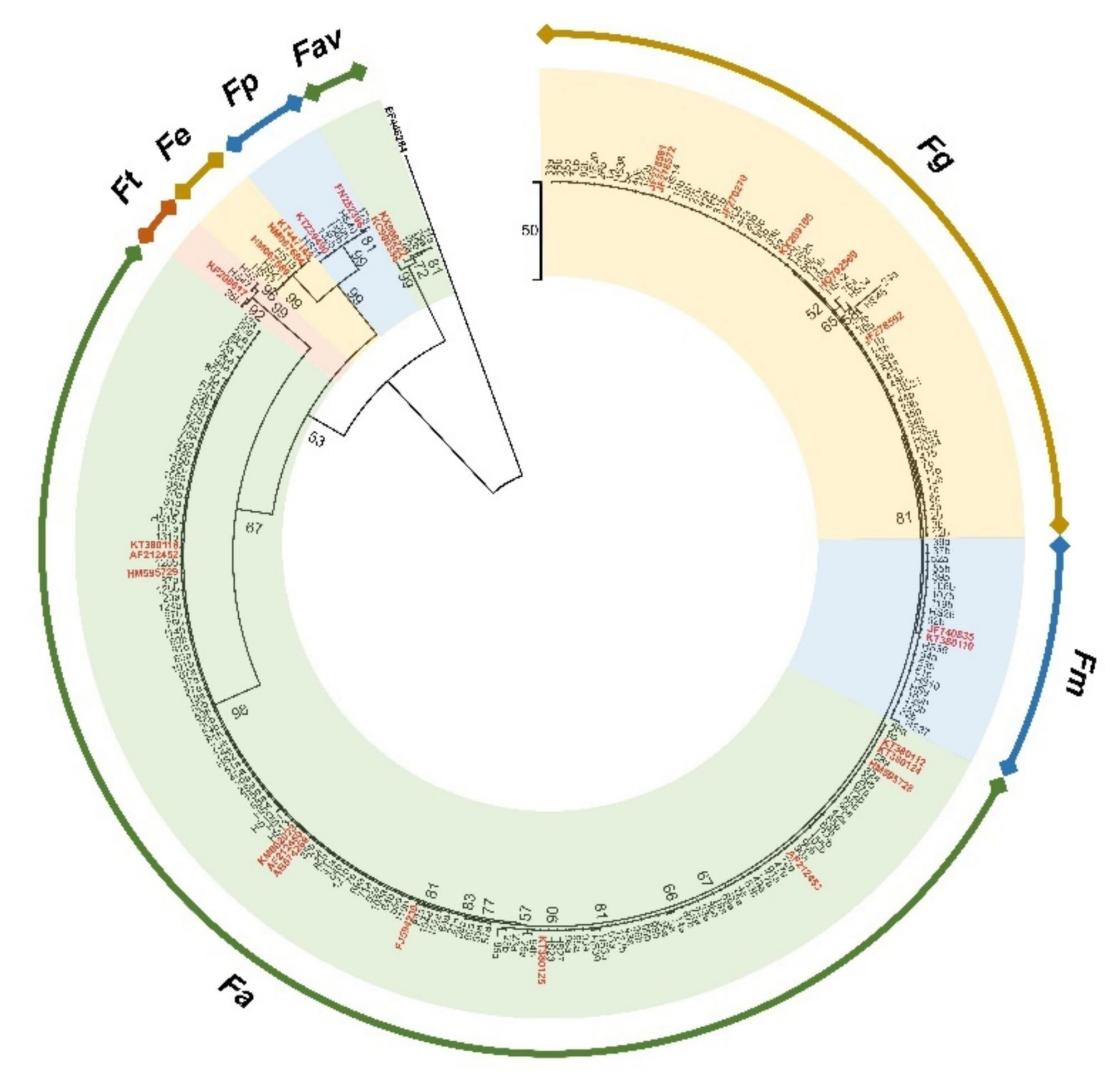
Phylogenetic tree of selected *Fusarium* isolates from FHB, wheat straw and maize stubble on the basis of *TEF1α* sequences. The dendrogram was constructed via the maximum parsimony approach and tested by bootstrapping (1000 replicates). The sequences of the isolates in bold were downloaded from the National Center for Biotechnology Information website (http://www.ncbi.nlm.nih.gov/BLAST). The reference strains used in this study are shown in red and bold. Fa: *Fusarium asiaticum*; **Fg**: *F. graminearum*; **Fm**: *F. meridionale*; **Fp**: *F. proliferatum*; **Fav**: *F. avenaceum*; **Ft**: *F. temperatum*; **Fe**: *F. equiseti*.

Using the species-specific primer pair Fg16F/Fg16R, 92 isolates were identified as *F. asiaticum*, accounting for 74.2%, and 32 isolates were *F. graminearum* sensu stricto (All subsequent references to *F. graminearum* in this text pertain to *Fusarium graminearum* sensu stricto), accounting for 25.8%. A maximum parsimony tree containing the remaining isolates and partial isolates subjected to specific PCR amplification was constructed on the basis of these *TEF1α* sequences (TL = 564, CI = 0.832, RI = 0.955, and RCI = 0.794), as shown in Fig. 2. In the phylogenetic tree, 234 *Fusarium* isolates were clustered into four clades with their corresponding species from GenBank. Those in the first clade, including all *F. asiaticum*, *F. graminearum* and *F. meridionale* isolates, belong to the FGSC. *F. equiseti* grouped into the second clade with a 99% bootstrap value. *F. temperatum*, and *F. proliferatum* were grouped into the third clade. Four isolates associated with FHB symptoms clustered in the *F. avenaceum* clade.

### *Fusarium* collection from wheat

On the basis of morphological and molecular analyses, 175 *Fusarium* isolates were identified as *F. asiaticum* (132 strains, 75.43%), *F. graminearum* (36 strains, 20.57%), *F. avenaceum* (4 strains, 2.29%), *F. meridionale* (2 strains, 1.14%) and *F. proliferatum* (1 strain, 0.57%) (Table 1). *F. asiaticum* and *F. graminearum* were the dominant species. *F. asiaticum* was found at each sampling location, but *F. avenaceum* was found only in Guang’an. *F. meridionale* and *F. proliferatum* were distributed only in Ziyang and Mianyang, respectively.

### Saprophytic *Fusarium* collection

Thirty-two strains were collected from maize stubble, and 104 strains were collected from wheat straw. Six *Fusarium* species were identified from maize stubble: *F. asiaticum* (10 strains, 31.25%), *F. graminearum* (10 strains, 31.25%), *F. meridionale* (5 strains, 15.63%), *F. temperatum* (3 strains, 9.38%), *F. equiseti* (2 strains, 6.25%) and *F. proliferatum* (2 strains, 6.25%). The isolates collected from wheat straw included five species: *F. asiaticum* (46 strains, 44.23%), *F. graminearum* (41 strains, 39.42%), *F. meridionale* (13 strains, 12.50%), *F. proliferatum* (3 strains, 2.88%) and *F. equiseti* (1 strain, 0.96%) (Table 1). Thus, the *Fusarium* composition of maize stubble and wheat straw differed at the same sampling site. *F. asiaticum* and *F. meridionale* were isolated from wheat straw, whereas *F. graminearum* was only isolated from maize stubble at the sampling site in Ya’an. In Chengdu, *F. proliferatum* was isolated from wheat straw but not maize stubble.

**Table 1:** The population composition of *Fusarium* isolates from different sources in a wheat‒maize cropping system.

Sources	FHB	Wheat straw	Maize stubble	Total
*F. asiaticum*	132	46	10	188
*F. graminearum*	36	41	10	87
*F. meridionale*	2	13	5	20
*F. proliferatum*	1	3	2	6
*F. avenaceum*	4	0	0	4
*F. equiseti*	0	1	2	3
*F. temperatum*	0	0	3	3
Total	175	104	32	311

### Pathogenicity assays

#### Pathogenicity of *Fusarium* spp. from wheat spikes

**Fig. 3:**
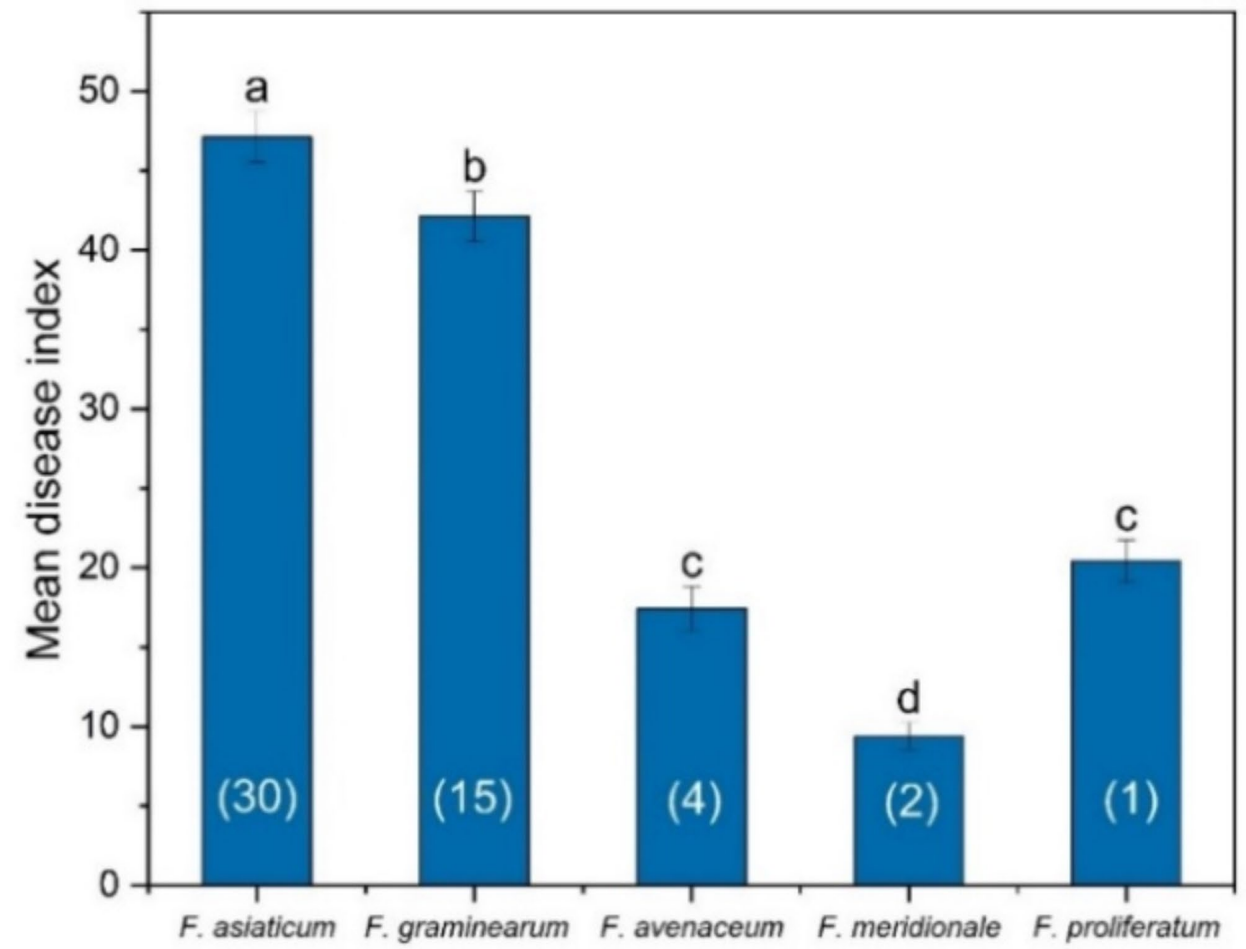
Pathogenicity of five *Fusarium* spp. from FHB in wheat. The wheat variety Chuanyu 20 was cultivated in a field that has been continuously planted with a wheat-maize rotation for at least six years. The numbers inside the parentheses are the numbers of strains. Columns with by the same letter do not differ significantly according to Fisher’s LSD test at *P* = 0.05.

All tested strains from wheat spikes caused apparent FHB symptoms, and the organisms were successfully reisolated from symptomatic tissues. No symptoms were observed on the control plants. The disease index varied among the different *Fusarium* species (Fig. 3). *F. asiaticum* had the greatest disease index (47.13), followed by *F. graminearum* (42.12). *F. asiaticum* and *F. graminearum* are considered highly pathogenic. The remaining three species with disease indices less than 21 were weakly pathogenic.

## Aggressiveness of the strains between *F. asiaticum* and *F. graminearum*

All the *Fusarium* strains tested for pathogenicity in the six wheat cultivars were able to produce FHB symptoms but showed variation in aggressiveness. Averaged across the six cultivars, the mean disease index of the *F. asiaticum* strains was greater than 45, indicating that it was highly aggressive, and no significant differences were found among the 5 strains (*P* > 0.05). The *F. graminearum* strains were less aggressive, with a mean disease index of less than 45. Furthermore, there was a significant difference among the *F. graminearum* strains (*P* < 0.05) (Table 2). The cultivars differed significantly regarding the main effect on the FHB disease index (*P* = 0.001). However, there was no significant two-way interaction effect between the cultivar and the *Fusarium* strains tested (*P* = 0.065) (Table 3).

**Table 2:** Disease index of six wheat cultivars inoculated with 5 strains of *Fusarium asiaticum* and 4 strains of *F. graminearum* from FHB.

*Fusarium* spp.	Isolate	Location	Disease index						
			Chuanyu 20	Mianyang 31	Chuanmai 104	Shumai 969	Sumai 3	Wangshuibai	Mean
*F. asiaticum*	4a	Shifang, Deyang	67.33	53.89	45.56	40	26.67	17.78	41.87 ± 7.35 ab
	8a	Jiangyou, Mianyang	54.22	57.78	48.33	53.33	31.67	26.11	45.24 ± 5.36 a
	20a	Shuangliu, Chengdu	55.2	63.89	60	50	27.78	24.44	46.89 ± 6.85 a
	39a	Wenjiang, Chengdu	63	61.11	52.67	47.78	29.44	21.67	45.95 ± 6.91 a
	69a	Jiangyou, Mianyang	68.89	75.56	58.89	51.11	33.89	25.56	52.32 ± 7.99 a
*F. graminearum*	1a	Yucheng, Ya’an	45.33	47.22	38.89	33.89	24.44	20.67	35.07 ± 4.43 abc
	5a	Yucheng, Ya’an	42	35	22.78	20	21.11	15.56	26.08 ± 4.15 bc
	10a	Renshou, Meishan	24.25	29.33	25	13.89	7.78	6.67	17.82 ± 3.94 c
	16a	Wenjiang, Chengdu	33.67	34	27.22	10.56	8.89	4.44	19.80 ± 5.44 c

Data are presented as mean ± standard deviation. Different lowercase letters in the same column indicate that there is a significant difference among the means, as determined by Fisher’s LSD test at *P* = 0.05.

**Table 3:** Disease indices of six wheat cultivars inoculated with 5 strains of *Fusarium asiaticum* and 4 strains of *F. graminearum* from FHB.

*Fusarium* spp.	Isolate	Location	Disease index						
			Chuanyu 20	Mianyang 31	Chuanmai 104	Shumai 969	Sumai 3	Wangshuibai	Mean
*F. asiaticum*	4a	Shifang, Deyang	67.33	53.89	45.56	40	26.67	17.78	41.87 ± 7.35 ab
	8a	Jiangyou, Mianyang	54.22	57.78	48.33	53.33	31.67	26.11	45.24 ± 5.36 a
	20a	Shuangliu, Chengdu	55.2	63.89	60	50	27.78	24.44	46.89 ± 6.85 a
	39a	Wenjiang, Chengdu	63	61.11	52.67	47.78	29.44	21.67	45.95 ± 6.91 a
	69a	Jiangyou, Mianyang	68.89	75.56	58.89	51.11	33.89	25.56	52.32 ± 7.99 a
*F. graminearum*	1a	Yucheng, Ya’an	45.33	47.22	38.89	33.89	24.44	20.67	35.07 ± 4.43 abc
	5a	Yucheng, Ya’an	42	35	22.78	20	21.11	15.56	26.08 ± 4.15 bc
	10a	Renshou, Meishan	24.25	29.33	25	13.89	7.78	6.67	17.82 ± 3.94 c
	16a	Wenjiang, Chengdu	33.67	34	27.22	10.56	8.89	4.44	19.80 ± 5.44 c

The wheats were cultivated in a field that has been continuously planted with a wheat-maize rotation for at least six years. The resistance levels of the varieties are as follows: “Mianyang 31” (susceptible), “Chuanyu 20” (moderately susceptible), “Chuanmai 104” (moderately susceptible), “Shumai 969” (moderately resistant), “Sumai 3”, and “Wangshuibai”. Data are presented as mean ± standard deviation. Different lowercase letters in the same column indicate that there is a significant difference among the means, as determined by Fisher’s LSD test at *P* = 0.05.

**Table 4:** Analysis of variance for the disease index of six wheat cultivars inoculated with 5 strarins of *Fusarium asiaticum* and 4 isolates of *F. graminearum*.

Sources of variation	Degrees of freedom	Mean square	F value	*P* value
Isolates	1	6313.543	126.045	0.001
Cultivars	5	1590.684	31.757	0.001
Isolates × Cultivars	5	113.522	2.266	0.065
Error	42	50.089		

**Table 5:** Analysis of variance for the disease indices of six wheat cultivars inoculated with 5 strains of *Fusarium asiaticum* and 4 isolates of *F. graminearum*.

Sources of variation	Degrees of freedom	Mean square	F value	*P* value
Isolates	1	6313.543	126.045	0.001
Cultivars	5	1590.684	31.757	0.001
Isolates × Cultivars	5	113.522	2.266	0.065
Error	42	50.089		

### Pathogenicity of saprophytic *Fusarium* species

**Fig. 4:**
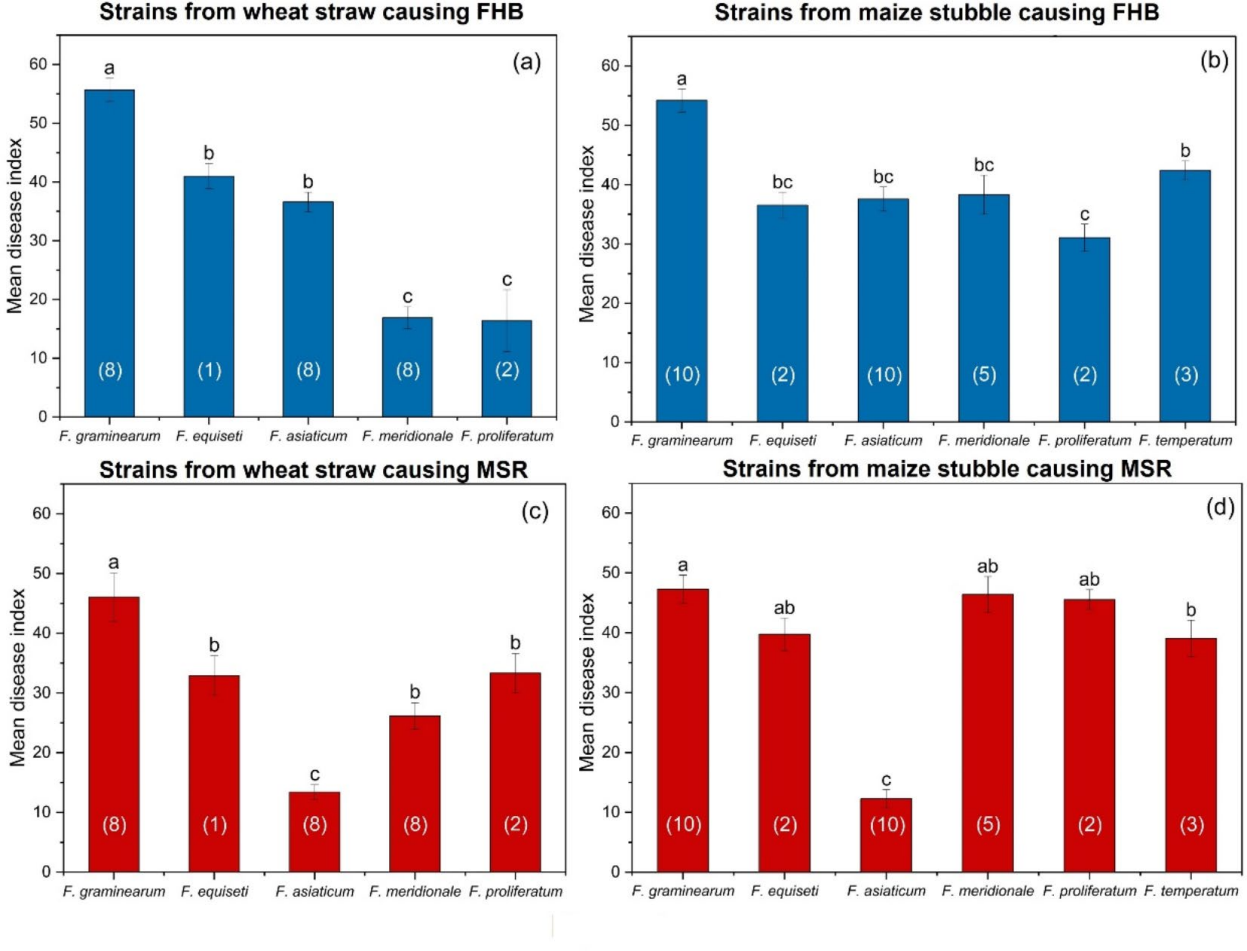
Pathogenicity in wheat and maize of saprophytic *Fusarium* spp. from wheat straw and maize stubble populations. (a): Strains from wheat straw causing FHB; (b): strains from maize stubble causing FHB; (c): strains from wheat straw causing MSR; (d): strains from maize stubble causing MSR. The wheat (variety Chuanyu 20) and maize (variety Chuandan 428) were cultivated in a field that has been continuously planted with a wheat-maize rotation for at least six years. The numbers inside the parentheses are the numbers of strains. Columns with by the same letter do not differ significantly according to Fisher’s LSD test at *P* = 0.05.

All the *Fusarium* strains from either the maize stubble or the wheat straw were able to cause FHB and MSR to different extents. Among the five *Fusarium* species isolated from wheat straw, *F. graminearum* was the most aggressive species for causing FHB, with a disease index of 55.67, followed by *F. equiseti* (40.97) and *F. asiaticum* (36.60). *F. meridionale* and *F. proliferatum* were relatively weakly aggressive in wheat, with disease indices of 16.91 and 16.37, respectively (Fig. 4a). Among the six *Fusarium* species isolated from maize stubble, *F. graminearum* was the most aggressive at causing FHB, with a disease index of 54.21, which was significantly greater than that of the other species. Moreover, the *F. temperatum*, *F. asiaticum*, *F. meridionale* and *F. equiseti* strains were highly aggressive, with disease indices greater than 36.00. *F. proliferatum* showed relatively weak aggressiveness, with a disease index of 31.07 (Fig. 4b).

Among the five species isolated from wheat straw, *F. graminearum* displayed the greatest ability to cause MSR, with a disease index of 46.07, followed by *F. proliferatum* (33.33), *F. equiseti* (32.92) and *F. meridionale* (26.16). *F. asiaticum*, with a disease index of 13.41, exhibited the weakest aggressiveness in maize (Fig. 4c). With disease indices ranging from 39.74 to 47.29, *F. graminearum*, *F. equiseti*, *F. meridionale* and *F. proliferatum* from maize stubble displayed high pathogenicity in maize. *F. asiaticum* was also the weakest pathogenic species, with a disease index of 12.27 (Fig. 4d).

**Fig. 5:**
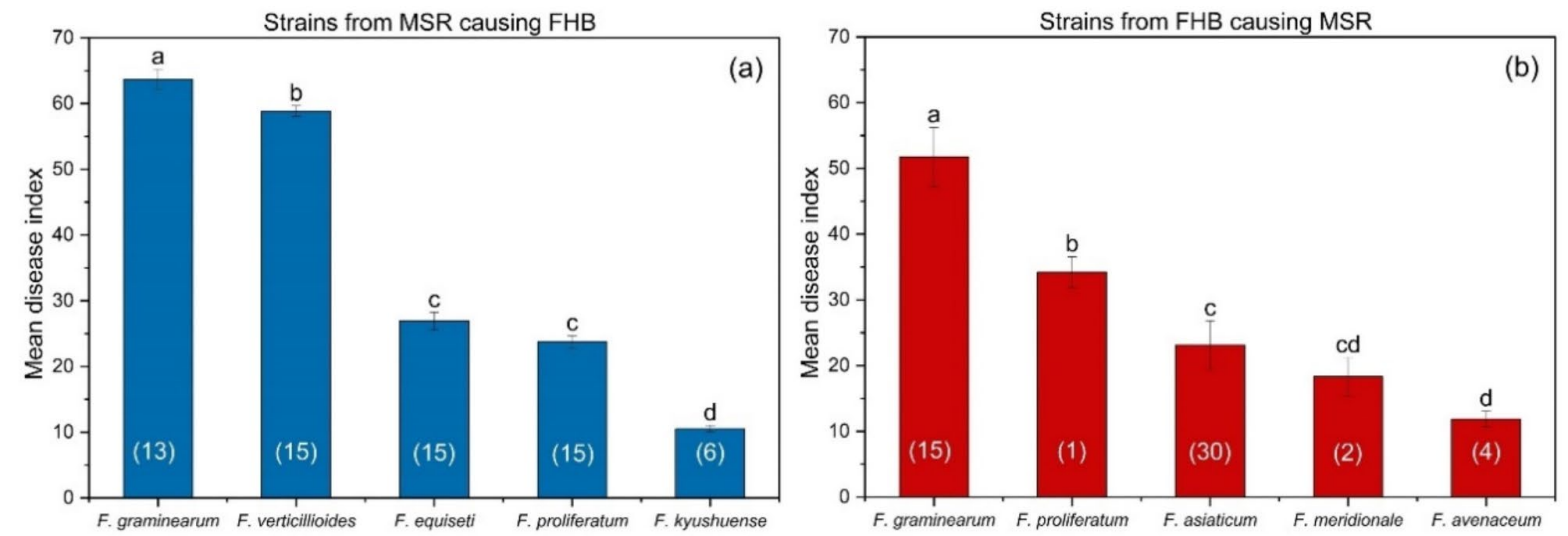
Cross-infections in wheat and maize of *Fusarium* spp. from FHB and MSR. (a) Strains from MSR causing FHB and (b) Strains from FHB causing MSR. Strains from wheat and maize (previously identified) were selected to cross-infect on maize (variety Chuandan 428) and wheat (variety Chuanyu 20). The wheat and maize were cultivated in a field that has been continuously planted with a wheat-maize rotation for at least six years. The numbers inside the parentheses are the numbers of strains. Columns with by the same letter do not differ significantly according to Fisher’s LSD test at *P* = 0.05.

### Cross-infection of *Fusarium* strains from FHB and MSR

Among the five *Fusarium* species from MSR, *F. graminearum*, with a disease index of 63.73, was the most aggressive species in wheat, followed by *F. verticillioides* (58.86). *F. equiseti* and *F. proliferatum* were less pathogenic, with disease indices of 26.94 and 23.77, respectively. With a disease index of 10.51, *F. kyushuense* was the species with the weakest effect on wheat (Fig. 5a). Among the five *Fusarium* species isolated from FHB, *F. graminearum* was the most aggressive species for infecting maize stems, with a disease index of 51.75, followed by *F. proliferatum* (34.20) and *F. asiaticum* (23.11). *F. meridionale* and *F. avenaceum* were relatively weakly pathogenic, with disease indices of 18.32 and 11.85, respectively (Fig. 5b).

## Discussion

FHB is the most severe fungal disease in wheat production. *Fusarium* species can not only infect a range of cereal crops during the growing season but also survive saprophytically on crop residues, where many ascospores released from perithecia serve as the primary inoculum for subsequent crops^11,29,30^. FHB affects a large area and has a negative effect on the yield and quality of wheat in China^5,6,8^. In the past, *Fusarium graminearum* complex was recognized as a single cosmopolitan species based on morphological identification; however, through genealogical concordance phylogenetic species recognition (GCPSR), this complex is now known to contain at least 11 distinct species. In order to enhance the scientific investigation of this pathogen and its associated diseases, *Fusarium graminearum* sensu stricto has undergone a redefinition^7,9,38–40^. At present, the *F. graminearum* complex encompasses 16 distinct species, with *F. graminearum* sensu stricto emerging as the most predominant species, succeeded by *F. asiaticum*, *F. meridionale*, *F. boothii*, and several other species^41^.

The diversity of FGSC pathogens associated with FHB is influenced by the host plant, geographical region, agroecosystem, and climatic conditions^6^. *Fusarium graminearum* is the predominant species on most wheat and other cereal crops; for instance, in South Africa, it is the dominant species on wheat and barley^42^. Over recent decades, the predominant species in Northern Europe has shifted from *F. culmorum* to *F. graminearum*, with changes potentially linked to reduced tillage, increased maize cultivation, or climatic factors^11,43^. *F. asiaticum* is better adapted to rice agroecosystems and is the most prevalent FGSC member on rice in Asia, particularly in Japan^20^, South Korea^44^, and Sichuan, China^45^. Zhang et al. have reported that crop rotation systems including rice can increase the prevalence of *F. asiaticum*, as observed in the Southwest and Middle-Lower Yangtze River regions where it is the predominant species. In contrast, *F. graminearum* dominates the Northeast and Huang-huai-hai regions^7^. Sichuan, situated in the upper reaches of the Yangtze River, has a humid and warm climate and is a moderately endemic area for FHB. In this region, wheat is commonly rotated with rice or corn, maintaining a planting area ratio of approximately 3:1 (wheat/rice or corn)^23^. In this study, we confirmed that *F. asiaticum* was the dominant species causing FHB in the wheat‒maize rotation system in Sichuan Province. Additionally, *F. graminearum* was frequently found, with an occurrence rate of 20.57% in wheat spikes. Prior research has indicated that a wheat-maize rotation tends to select for *F. graminearum*, while a wheat-rice rotation favors the selection of *F. asiaticum*^6,23,31,45,46^. However, our study revealed that the prevalence of *F. graminearum* is significantly higher in Sichuan compared to other provinces in the Yangtze River Basin, a finding consistent with the results reported by Zhang et al.^7^. Concurrently, *Fusarium meridionale* demands our attention due to the quantity of isolates obtained. *F. meridionale* has previously been reported on wheat and barley but typically occurs at low frequencies^7,42^. In Nepal, Brazil and northern Argentina, *F. meridionale* is the predominant species on maize^47–49^, and small populations of *F. meridionale* have also been reported in China and South Korea^21,50^. A report on the FGSC associated with FHB in wheat in Brazil found that, in addition to *F. graminearum*, there is a substantial presence of *F. meridionale*, which is believed to be related to the twice-yearly maize harvests, and also attributed to climate changes that allow subdominant species to play a significant role in years less favorable for *F. graminearum*^15,51^. In this study, the isolation frequency of *F. meridionale* was second only to the two dominant species, consistent with previous isolation results^31,45^. The wheat‒maize cropping system is more prevalent in Sichuan than in other provinces in this region. Therefore, an important reason may be the pressure by maize in rotation.

In the present study, *F. asiaticum*, *F. graminearum*, *F. meridionale*, and *F. proliferatum* are common species in this wheat-maize cropping system, isolated from all sources, including wheat spikes, wheat straw, and maize stubble. This result is fundamentally consistent with the group and ratio obtained from Zhang’s 2023 survey on wheat FHB pathogens in Sichuan over the past two years^52^. *F. asiaticum* may be the dominant pathogen of FHB, whereas *F. graminearum* is the dominant pathogen of MSR. In the whole wheat‒maize rotation system, the *Fusarium* population is forced to undergo two selection cycles (wheat or maize) every year, which ultimately leads to significant competition between *F. asiaticum* and *F. graminearum* during both the infection stage and the saprophytic stage.

However, *F. avenaceum* was isolated only from spikes. *F. equiseti* and *F. temperatum* were present only at the saprophytic stage. Our data indicate that the *Fusarium* population of saprophytic sources was more diverse than that of infected kernels. Both *F. asiaticum* and *F. graminearum* from saprophytic sources were dominant, with almost equal isolation frequencies. *F. temperatum*, which is closely related to *F. subglutinans*, has previously been reported as a new biological species causing maize ear rot in Jilin, Hubei, and Guizhou in China^53^. Nonetheless, this study is the first report of *F. temperatum* on maize stubble in Sichuan. *F. temperatum* was highly pathogenic in the pathogenicity assays, indicating that it is a potential threat to maize and wheat.

*Fusarium* species not only impair yield and reduce quality but are also notorious for their ability to produce trichothecenes, which pose a significant threat to human and animal health. The most important B-type trichothecene mycotoxins produced are nivalenol (NIV), deoxynivalenol (DON), and the acetylated compounds 3-acetyl deoxynivalenol (3-AcDON) and 15-acetyl deoxynivalenol (15-AcDON)^11^. *Fusarium* species vary in their mycotoxin production profiles. Specifically, chemotypes of *F. graminearum* predominantly produce DON, with 15-AcDON and 3-AcDON as secondary metabolites, and NIV is exceptionally rare. In contrast, *F. asiaticum* is more often associated with the NIV chemotype, followed by the DON chemotype and its acetylated chemotypes. *F. meridionale* is found almost exclusively in the NIV chemotype^41^. Variations in the chemotype composition of trichothecene-producing *Fusarium* species are significantly influenced by factors including region, host type, climatic characteristics, and agricultural systems, with variations occurring annually^34,46^. Ward et al. (2008) found that the 3-AcDON chemotype of *F. graminearum* in western Canada is rapidly replacing the predominant 15-AcDON chemotype. In the east, all isolates are *F. graminearum* producing 3-AcDON mycotoxins, while in the west, *F. graminearum* producing 3-AcDON mycotoxins accounts for less than 10%. Furthermore, between 1998 and 2004, the population of *F. graminearum* producing 3-AcDON mycotoxins in the west increased fourteenfold^12,13^. Population analyses in Brazil revealed that *F. meridionale* and *F. cortaderiae* with the NIV chemotype are significant regional contributors to FHB, due to the extensive cultivation of maize in the area^15,51^. Maize is the most important cereal crop in northwestern Iran, while rice predominates in the northeast, leading to a shift from 15-AcDON-producing isolates in the west to NIV-producing isolates in the east^54^. Similarly, in Europe, with the increase in maize cultivation, *F. graminearum* has become the dominant pathogen, and the type of mycotoxins it produces has shifted from NIV to 15-AcDON^11^. In the Yangtze River Basin and the regions to its north, the dominant chemotypes are 3-AcDON and 15-AcDON^22^. In the lower reaches of the Yangtze River, particularly in Hefei and Yangzhou, DON is the predominant mycotoxin found, whereas in Nanping, Fujian—where wheat is scarcely cultivated and rice predominates—the primary mycotoxin detected is NIV^23^. Furthermore, our research indicates that rice serves as the preferred ecological niche in Japan^20^, South Korea^44^, and Sichuan Province, China^7^. Within these regions, the NIV chemotype of *F. asiaticum* is more prevalent^23^. It is evident from the above that the geographical distribution of chemotypes correlates with temperature variations and crop rotation practices^22^. Host preference is likely the principal factor contributing to the widespread prevalence of the NIV chemotype of *F. asiaticum* in rice-wheat rotation fields. In this study, we utilized specific primers TOXP1/P2, Tri303F/R, and Tri315F/R to assess the trichothecene chemotypes of dominant *Fusarium* isolates from wheat scab, including 31 isolates of *F. graminearum* and 103 isolates of *F. asiaticum*^22^. The results revealed that the NIV chemotype was predominant (76.12%). Significant differences were observed in the chemotype composition between these two *Fusarium* species. In *F. graminearum* isolates, chemotypes 15-AcDON (77.4%) and NIV (22.6%) were detected, whereas in *F. asiaticum*, chemotypes 15-AcDON (3.88%), 3-AcDON (3.88%), and NIV (92.24%) were identified (data not shown). Yan (2022) suggests that the NIV chemotype is more prevalent in rice-wheat rotation systems than in maize-wheat rotation systems^23^. In our study, the NIV chemotype of *F. asiaticum* was found to be predominant. The distribution of chemotypes is also associated with geographical location and strain variability, which is consistent with the findings of Yang et al.^45^. We hypothesize that the extensive cultivation of rice in the Sichuan region is a primary factor, further compounded by the geographical constraints of the region’s hilly and mountainous terrain, and additionally, the rise in labor costs, has contributed to a gradual reduction in wheat cultivation, thus limiting the spread of various *Fusarium* species on wheat. Consequently, the selective pressure for *Fusarium* species with higher aggressiveness on wheat has also been reduced. Furthermore, in this cropping system, the cultivation of maize is a significant factor associated with the higher isolation frequency of *F. graminearum*, with its NIV chemotype reaching 22.6%, a proportion significantly higher than that observed in the middle and lower reaches of the Yangtze River and the Huang-Huai-Hai regions^7,23,45^.

Mycotoxins, as significant pathogenic factors, exert multiple effects during their interaction with hosts, such as inducing cell necrosis, inhibiting growth, and suppressing seed germination^11,41^. However, the correlation between mycotoxin type and disease severity remains elusive. Studies conducted on *F. graminearum* isolates from various regions of the United States and Canada report that 3-AcDON isolates demonstrate higher aggressiveness towards wheat than 15-AcDON isolates^55^. Conversely, Kuhnem et al. (2015) observed in New York that the 3-AcDON chemotype of *F. graminearum* displays substantial differences in aggressiveness between wheat and maize in comparison with 15-AcDON isolates, showing no significant correlation^56^. Yan et al. (2022) observed a significant correlation between DON and disease index across major wheat-producing regions of China following natural disease outbreaks. Different isolates of the same species display variability in mycotoxin chemotypes, which correlates with differences in aggressiveness. Generally, species with the DON chemotype exhibit higher aggressiveness to wheat than those with the NIV chemotype^23^. *F. asiaticum* (NIV chemotype) is a predominant pathogen associated with FHB in Sichuan, with its aggressiveness significantly lower compared to *F. asiaticum* (3-AcDON chemotype) and *F. graminearum* (15-AcDON chemotype), which are predominant in the middle and lower reaches of the Yangtze River^7,22,45^. Additionally, Del Ponte (2015) in Brazil observed that the *F. graminearum* (15-AcDON chemotype) exhibit greater competitiveness on wheat compared to the *F. meridionale* (NIV chemotype)^51^. In this study, *F. graminearum*, irrespective of their origin from maize stubble or wheat straw, result in the highest disease indices for MSR and FHB, whereas *F. asiaticum* exhibits the lowest disease index for MSR among all strains. This indicates that *F. asiaticum* possesses higher aggressiveness on wheat and is better adapted to wheat as a host. The aggressiveness of *F. graminearum* on wheat exceeds that of the predominant isolates *F. asiaticum*, which we attribute to the fact that the majority of *F. graminearum* isolates possess the 15-AcDON chemotype, whereas *F. asiaticum* isolates possess the NIV chemotype. This difference in chemotype is believed to contribute to the varying levels of aggressiveness observed between the two strains on wheat.

Furthermore, *F. meridionale* isolates from wheat straw and infected spikes exhibit lower FHB disease indices compared to those from maize stubble. The majority of *F. meridionale* chemotypes are NIV, and have been reported to be primarily associated with maize^41,51^. Although we have not yet tested the chemotype of *F. meridionale*, we hypothesize that its aggressiveness on maize is maintained, whereas the reduced aggressiveness on wheat is likely attributed to its minor and limited population. Furthermore, wheat has only recently been recognized as a host for *F. meridionale*, and still requires further adaptation. This suggests that the species may still be in the process of adapting to wheat, which could explain the observed differences in aggressiveness between the two hosts. Ultimately, other species demonstrate significant aggressiveness on both maize and wheat. Xu et al. (2021) reported that temperature and cultivation patterns influence the distribution of *F. graminearum* and *F. asiaticum*^46^. Based on these findings, we hypothesize that in regions or years with unfavorable weather conditions for FHB outbreaks, less dominant pathogenic strains may play a more substantial role.

Some studies indicate substantial variability in the correlation between DON and disease severity. This variability is likely due to several factors that modulate this relationship, including the disparate rates of DON production by *F. graminearum* isolates, the resistance of cultivated varieties to FHB and DON accumulation, and the weather conditions that facilitate infection and growth of *F. graminearu*^57^. Additionally, literature review reveals that disease index assessments are commonly determined by visually estimating lesion diameter, whereas in wheat, the focus is on evaluating the PSS (proportion of symptomatic spikelets in a spike). Occasionally, there is a lack of significant differences between grades or a delay in symptom expression, which inevitably amplifies the likelihood of diagnostic errors due to human subjectivity^23,56,58^.

Pathogenicity tests frequently induce a robust immune response from the host’s primary effector genes, culminating in qualitative resistance and subsequent necrotic responses at the inoculation sites. The mechanisms by which necrotic responses, a manifestation of qualitative resistance, correlate with quantitative resistance—encompassing the pathogenicity or aggressiveness of the fungus and the resistance of the plant—remain elusive^59^. The application of quantitative PCR (qPCR) allows for the inference of fungal biomass quantification by detecting the copy number of housekeeping genes, or the employment of strain-specific primers to identify various pathogen categories, elucidates the pathogenic potential of fungal strains at both macroscopic and microscopic scales^60^. Scauflaire et al. (2012) developed a qPCR assay utilizing hybridization probes for the specific identification, detection, and quantification of four mycotoxin-producing *Fusarium* species in maize, sensitive enough to detect *Fusarium* DNA at concentrations as low as 5 pg in single plex and 50 pg in multiplex formats^61^. Bilska et al. (2018) devised a highly sensitive qPCR detection approach based on mitochondrial DNA, enabling the reliable quantification of *F. culmorum* fungal DNA down to 0.01 pg^62^. Sherif (2023) employed qPCR to track the biomass of *F. graminearum* and *F. verticillioides* in vitro co-cultures and plant co-infections. The study observed that *F. graminearum* could enhance the growth of *F. verticillioides*, wherein fumonisin exerted an effect in mitigating competitive interactions among *F. verticillioides*^63^. Wang (2023) aimed to ascertain the pathogenic role of *Fg62* in *F. graminearum* by employing qPCR to assess biomass in wheat stems post-infection, revealing that the absence of *Fg62* led to shorter lesion lengths and a reduced fungal establishment^64^. The population structure and species distribution of FGSC in the North China Plain and the Yangtze River Basin, as determined by the developed qPCR assays, were consistent with previous results obtained using the fungal isolation method^65^. It is evident that qPCR, owing to its heightened sensitivity, accurate identification, and precise quantification, more accurately reflects the pathogenic potential of fungi towards their hosts, addressing the shortcomings of direct visual estimation in providing precise measurements. Regrettably, our study, in line with previous research, did not utilize these sophisticated measurement techniques, opting instead for the proportion of symptomatic spikelets (PSS) as a basis for assessment.

Furthermore, beyond disease indices, the accumulation of mycotoxins is a critical post-colonization trait of *Fusarium*, which is also associated with the crop harvest^66^. Nonetheless, a definitive correlation between disease indices and mycotoxin accumulation remains elusive. Ward et al. (2008) proposed that mycotoxins accumulation does not correlate with the disease indices^12^. In contrast, Von der Ohe et al. (2010) established that 3-AcDON strains substantially outpace 15-AcDON strains in DON accumulation within wheat, despite similar disease indices^67^. Mycotoxin contamination levels, influenced by environmental factors and annual variations, fluctuate in accordance with standard climatic conditions^66^. Yan et al. (2022) conducted research demonstrating a positive correlation between disease indices and DON accumulation, alongside significant variations in resistance to FHB and mycotoxin accumulation among diverse regions, climatic conditions, and wheat cultivars. Notably, temperature and rainfall emerge as pivotal climatic variables^23^.

A recent inoculation experiment, utilizing a variety of methodologies, disclosed an absence of a necessary correlation between disease severity and DON accumulation^58^. Malihipour’s study disclosed significant variations in pathogenicity among *Fusarium* strains and in the responses of wheat genotypes, exhibiting consistent infectivity across various susceptible hosts, as well as significant resistance in genotypes G-14 (SHA3/CBRD) and G-15 (NG8675/NING8645), suggesting their potential as stable resistance sources^68^. This implies that the presence of stable resistance genes can effectively suppress disease severity and mycotoxin accumulation, keeping them at minimal levels. In our study, we selected five strains of *F. asiaticum* and four strains of *F. graminearum* for pathogenicity assays on wheat cultivars exhibiting varying degrees of resistance. Differences in disease indices were directly observable between resistant and susceptible cultivars. Aggressiveness variations among different strains of *F. asiaticum* on the same cultivar were not significant; in contrast, distinct strains of *F. graminearum* exhibited significant differences. Additionally, for wheat cultivars with equivalent resistance levels, disease indices for various strains of *F. asiaticum* were higher than those of *F. graminearum*, particularly in susceptible cultivars. Subsequently, a chemical profiling analysis of the strains employed in the pathogenicity trials revealed minimal variation in disease indices among *F. asiaticum* strains of the same (NIV) or differing chemical types (NIV, 3AcDON, 15AcDON) across wheat with varying resistance levels, with the NIV chemotype manifesting the highest disease index in susceptible cultivars. Consistently, no correlation was observed between disease indices and the chemical types of *F. graminearum* post-infection in wheat.

Our evaluation of disease indices in this experiment relied on visual estimation, a method that inherently carries a certain degree of inaccuracy. Additionally, we did not perform assessments for fungal biomass or mycotoxin accumulation, and our findings were potentially confounded by variations in inoculation conditions and climatic factors. Consequently, our preliminary findings indicate that there is no direct correlation between chemical types and disease indices. The interplay among different chemical types, disease indices, and mycotoxin accumulation merits thorough investigation in subsequent studies.

The wheat-maize rotation system, prevalent in Sichuan’s distinct climatic and geographic context, effectively reduces the reliance on chemical inputs and enhances grain yield^25–28^. However, this agricultural practice may intensify the aggressiveness of *Fusarium* species, underscoring the need for continuous monitoring of these pathogens^30^. Our study has identified significant *Fusarium* species, including *F. asiaticum* and *F. graminearum*, within the wheat-maize fields of Sichuan, capable of maintaining cyclic infections that result in FHB and MSR. Therefore, the management of crop residues is pivotal in mitigating disease prevalence. Importantly, wheat exhibits markedly higher contamination levels of NIV and DON mycotoxins compared to corn and rice, with the NIV chemotype being predominant and a significant contributor to toxin contamination^70^. Although no distinct correlation was observed between toxin types and disease indices, variations in aggressiveness among different species were noted. Future breeding programs aimed at resistance must take into account the distribution of toxins within *Fusarium* populations and screen for resistance against a spectrum of highly aggressive strains. Elucidating the relationship between FHB disease indices and mycotoxin accumulation is essential for resistance breeding and for the post-harvest surveillance of toxin concentrations in grain and feedstuffs.

## Conclusion

The wheat‒maize cropping system is a prevalent cultivation pattern in Sichuan Province, China. In this system, FHB has become increasingly severe, such that *Fusarium* rot disease is also becoming a severe threat to maize. Here, we present the first report of the population composition and aggressiveness of *Fusarium* species present in naturally infected wheat spikelets and crop residues in a wheat‒maize cropping system in Sichuan Province. *F. asiaticum* and *F. graminearum* emerge as the predominant species, characterized by NIV and 15AcDON chemical types, respectively, yet their pathogenicity varies with different chemotypes. The intricate relationship between toxin chemotypes and disease indices suggests the involvement of additional determinants. Our findings underscore the necessity for future research to thoroughly investigate the interplay among chemical types, disease indices, and mycotoxin accumulation, as well as their implications for crop resistance and susceptibility. Such focused research is essential for deepening our comprehension of *Fusarium* head blight and for devising precise control strategies within the region’s agricultural practices.

## Materials and methods

### Sample collection and isolation

Samples of typical wheat spikes characterized by clear symptoms of FHB were collected during the wheat growing season from 16 different administrative districts throughout the wheat‒maize cropping regions in Sichuan Province, China. Furthermore, samples of wheat straw exhibiting black-colored *Fusarium* perithecia were collected during the maize jointing stage, and samples of maize stubbles exhibiting black-colored *Fusarium* perithecia were collected during the wheat elongation stage from the same sampling sites distributed in five wheat‒maize cropping regions: Chengdu, Ya’an, Deyang, Mianyang, and Meishan in Sichuan Province. All the sampling sites were distributed in different villages or fields. The samples from each site were investigated via a five-point sampling method. For each sample, at least ten affected wheat spikes, straws or stubble from each sample were collected. All the samples were air dried at room temperature (25 to 30 °C) for 24 h and then stored at − 20 °C until processing.

The spikelets were cut into pieces 3–4 mm in length, surface sterilized in 75% ethanol (V/V) for 3 min, followed by 0.5% NaOCl (W/V) for 2 min, and then rinsed three times with sterilized distilled water. After drying on sterile filter paper, the spikelets were placed onto potato dextrose agar (PDA) and incubated for 4 days at 25 °C. Mycelia were subsequently inoculated on PDA or carboxymethyl cellulose (CMC) media to produce spores. Single-spore isolates from wheat spikes were finally acquired via the method described by Qu et al.^19^ Maize stubbles and wheat straws exhibiting perithecia were cut into small segments. The surfaces were disinfected in 75% ethanol (V/V) for 2 min and 0.5% NaOCl (W/V) for 30 s, rinsed three times with sterilized distilled water, and blotted dry on sterile filter paper. Under a microscope, a single perithecium was removed from the segment and crushed for single-ascospore isolation. Single ascospore was purified via the method of Pereyra^71^ and Dill-Macky^36^. One or two isolates were derived from each sampling location. The colony characteristics and spore morphology of these single-spore isolates on PDA or CMC were observed and recorded according to the methods described by Leslie and Summerell^72^.

### DNA extraction

Each isolate was cultured on PDA at 27 °C for 3‒5 d, and genomic DNA was extracted from mycelia via the cetyltrimethylammonium bromide (CTAB) method^73^. The DNA concentration and quality were estimated via a NanoDropTM 2000 spectrophotometer (Thermo Fisher Scientific, Delaware, USA). The DNA was diluted to 50 ng/µL and stored at -20 °C.

### PCR amplification and sequencing

PCR amplification was carried out in a final volume of 20 µL containing 1 µL of genomic DNA (approximately 50 ng), 0.5 µL of each primer (10 µM), 10 µL of Taq PCR Master Mix (Sangon Biotech, Shanghai, China), and 8 µL of ddH2O. The primer pairs Fg16F (5’-CTCCGGATATGTTGCGTCAA-3’) and Fg16R (5’-GGTAGGTATCCGACATGGCAA-3’) were used for the detection of *F. asiaticum* and *F. graminearum* described by Nicholson et al.^74^. The cycling parameters were 95 °C for 5 min, followed by 35 cycles of 45 s at 95 °C, 30 s at 55 °C, and 1 min at 72 °C and 10 min at 72 °C^38^. The *F. graminearum* isolate amplicon is 410 bp, and the *F. asiaticum* amplicon is 497 bp. The translation elongation factor 1α (*TEF1α*) sequence was amplified via the primer pair EF-1 (5’-ATGGGTAAGGAGGACAAGAC-3’) and EF-2 (5’-GGAAGTACCAGTGATCATG-3’)^75^. The PCR conditions were as follows: initial denaturation for 2 min at 96 °C; 35 cycles of 30 s at 94 °C, 30 s at 53 °C, and 30 s at 72 °C; and a final extension for 10 min at 72 °C. The PCR products were assessed via 1.5% agarose gel electrophoresis. *TEF1α* sequences were obtained from Sangon Biotech Co., Ltd. (Shanghai, China). All *TEF1α* sequences of the *Fusarium* isolates obtained in this study were deposited in GenBank.

### Phylogenetic analysis

The *TEF1α* sequences from the *Fusarium* isolates were compared to sequences in the National Center for Biotechnology Information (NCBI) database via BLASTn (BLAST network services) and then aligned together with reference sequences obtained from GenBank via Clustal X v2.0, with characters weighted equally. Phylogenetic trees were constructed in MEGA 6.0 via the maximum parsimony approach based on the Kimura 2-parameter model, rooted with *Bipolaris oryzae* (KJ939510) as the outgroup. The bootstrap values provided on the phylogenetic dendrogram were generated with 1000 replicates, and alignment gaps were excluded. In addition, descriptive tree statistics, such as parsimony (Tree Length [TL], Consistency Index [CI], Retention Index [RI], and Related Consistency Index [RCI]), were calculated.

### Plant materials

Six winter wheat cultivars with different levels of resistance to FHB and one maize cultivar were used. “Mianyang 31” (susceptible), “Chuanyu 20” (moderately susceptible), “Chuanmai 104” (moderately susceptible) and “Shumai 969” (moderately resistant) are the main cultivars in Sichuan, and the resistance of these cultivars to FHB has been evaluated in our laboratory for several years. “Sumai 3” (resistant) and “Wangshuibai” (resistant) are the most widely used sources of FHB resistance worldwide^3^. One maize cultivar, “Chuandan 428” (susceptible) was used, which is a primary maize cultivar in Sichuan. Each wheat cultivar was planted in three blocks (2 m × 4 m per block) according to standard agronomic practices at Wenjiang Farm (wheat‒maize continuously planted for a minimum of 6 years) in Chengdu (the experimental farm of Sichuan Agricultural University). After the wheat was harvested, maize was planted in three blocks (4 m × 6 m per block) according to standard agronomic practices. The experiment involved a randomized complete block design with three replicates.

### Preparation of *Fusarium* inoculums

A conidial suspension was prepared by scraping mycelia into Czapek-Dox media and shaking at 120 rpm for 6 d at 27 °C. The suspension was filtered with layers of cheesecloth and adjusted under a microscope to a concentration of 1 × 10^5^ conidia in 1 mL of sterile water containing 0.01% Tween 20.

### Pathogenicity assays

Four experiments were carried out to evaluate the aggressiveness of these *Fusarium* strains: (i) 30 strains of *F. asiaticum*, 15 strains of *F. graminearum* and all *F. avenaceum* (4 strains), *F. meridionale* (2 strains), and *F. proliferatum* (1 strain) from spikes were used to test the pathogenicity to wheat plants (variety Chuanyu 20). (ii) 5 representative strains of *F. asiaticum* and 4 strains of *F. graminearum* from wheat were selected to evaluate the aggressiveness to six wheat cultivars (variety Chuanyu 20, Mianyang 31, Chuanmai 104, Shumai 969, Sumai 3 and Wangshuibai). (iii) 27 strains from wheat straw and 32 strains from maize stubbles were selected to test the pathogenicity in wheat (variety Chuanyu 20) and maize (variety Chuandan 428), respectively. And (iv) 52 strains from wheat and 64 strains from maize (previously identified) were selected to cross-infect on maize (variety Chuandan 428) and wheat (variety Chuanyu 20).

During the inoculation process of wheat, a precise single-floret injection technique was employed to assess the pathogenicity of the samples in wheat^45^. Twenty spikes at the 50% anthesis stage were randomly selected from each block for treatment with each representative strain. Twenty microliters of conidial suspensions were injected into the central spikelet. In addition, twenty spikes from each block were inoculated with 20 µL of sterile water as controls. After inoculation, each wheat spike was covered with a plastic bag for 72 h to maintain humidity. Pathogenicity was assessed as the percentage of infected spikelets at 21 days after inoculation. Disease index was calculated based on the proportion of symptomatic spikelets (PSS) to assess disease severity, categorized on a 0–4 scale^76^. Disease index =(∑(A×B))/(∑B×4) ×100, where A represents the disease scale (0, 1, 2, 3, or 4) and where B represents the number of plants at each disease level.

During the inoculation of maize, a channel inoculation method was used to inoculate the maize stems. The penultimate stem internode section was pricked with an obtuse needle, and 1 mL of conidial suspension was injected into the wounded tissue at the tasseling stage via a sterile syringe. Maize stems inoculated with sterile water were used as negative controls. Fifteen maize plants were inoculated with each strain in each block in a randomized design. Disease levels were assessed on day 20 after inoculation. Each inoculated maize stem was divided, and the MSR severity was rated on a scale of 1–9, as described in Zhang et al.^76^ Disease index = (∑(A×B))/(∑B×9) ×100, where A represents the disease scale (1, 3, 5, 7, or 9), and B represents the number of plants at each disease level.

### Statistical analyses

The aggressiveness of *Fusarium* strains on wheat and maize was analyzed via one-way analysis of variance (ANOVA). The data were tested for a normal distribution and equal variance prior to ANOVA. When ANOVA indicated a significant difference between treatments (*P* < 0.05), the treatment data were compared via Fisher’s least significant difference (LSD) test. The data of the nine *F. asiaticum* and *F. graminearum* strains tested in the six cultivars were assessed for homogeneity of variance via Levene’s test. The variability in the observations in the experiments for the disease index was comparable, and an ANOVA could be validly carried out. Fisher’s LSD was calculated at the 5% level to determine the significance differences in the treatment means. All analyses were performed with IBM SPSS Statistics v21.0 (Armonk, New York, USA).

## Electronic Supplementary Material

Below is the link to the electronic supplementary material.

Supplementary Material 1

## Data Availability

The data generated during this study are available upon reasonable request to the corresponding author.
