# Supplementary material for: Diversity and pathogenicity of Fusarium species associated with Fusarium head blight in wheat and maize cropping systems in Sichuan Province
Source: Sci Rep. 2025 Feb 18;15:5984. doi: 10.1038/s41598-024-83402-7 (PMC11836280; doi:10.1038/s41598-024-83402-7)
Supplement: Supplementary file 1 — Supplementary Material 1 [file 41598_2024_83402_MOESM1_ESM.pdf]

Table S1 Information of *Fusarium* species isolated from wheat spikes, wheat straws and maize stubbles collected from different wheat-maize cropping regions in Sichuan, China

Isolate	Source	Location	GenBank accession number of *TEF1α*	Suggested identification
1a	wheat spike	Yucheng, Ya'an	KY283854	*F. graminearum*
2a	wheat spike	Shehong, Suining	KY283855	*F. asiaticum*
3a*	wheat spike	Lezhi, Ziyang	KY283856	*F. asiaticum*
4a	wheat spike	Shifang, Deyang	KY283857	*F. asiaticum*
5a*	wheat spike	Yucheng, Ya'an	KY283858	*F. graminearum*
6a	wheat spike	Dujiangyan, Chengdu	KY283859	*F. graminearum*
7a	wheat spike	Shehong, Suining	KY283860	*F. asiaticum*
8a*	wheat spike	Jiangyou, Mianyang	KY283861	*F. asiaticum*
9a	wheat spike	Tianquan, Ya'an	KY283862	*F. asiaticum*
10a*	wheat spike	Renshou, Meishan	KY283863	*F. graminearum*
11a	wheat spike	Linshui, Guang'an	KY283864	*F. avenaceum*
12a	wheat spike	Linshui, Guang'an	KY283865	*F. avenaceum*
13a	wheat spike	Xichong, Nanchong	KY283866	*F. asiaticum*
14a	wheat spike	Anzhou, Mianyang	KY283867	*F. asiaticum*
15a*	wheat spike	Anzhou, Mianyang	KY283868	*F. asiaticum*
16a	wheat spike	Wenjiang, Chengdu	KY283869	*F. graminearum*
17a	wheat spike	Xichong, Nanchong	KY283870	*F. proliferatum*
18a	wheat spike	Tianquan, Ya'an	KY283871	*F. asiaticum*
19a	wheat spike	Xuanhan, Dazhou	KY283872	*F. asiaticum*
20a*	wheat spike	Shuangliu, Chengdu	KY283873	*F. asiaticum*
21a*	wheat spike	Shuangliu, Chengdu	KY283874	*F. asiaticum*
23a*	wheat spike	Shifang, Deyang	KY283875	*F. asiaticum*
24a	wheat spike	Shehong, Suining	KY283876	*F. graminearum*
25a*	wheat spike	Lezhi, Ziyang	KY283877	*F. asiaticum*
26a	wheat spike	Shuangliu, Chengdu	KY283878	*F. asiaticum*
27a*	wheat spike	Jiangyou, Mianyang	KY283879	*F. asiaticum*
28a	wheat spike	Shuangliu, Chengdu	KY283880	*F. asiaticum*
29a	wheat spike	Linshui, Guang'an	KY283881	*F. avenaceum*
30a	wheat spike	Linshui, Guang'an	KY283882	*F. avenaceum*
31a	wheat spike	Longchang, Neijiang	KY283883	*F. asiaticum*
32a	wheat spike	Zizhong, Neijiang	KY283884	*F. asiaticum*
33a*	wheat spike	Shuangliu, Chengdu	KY283885	*F. asiaticum*
35a	wheat spike	Dongpo, Meishan	KY283886	*F. asiaticum*
36a*	wheat spike	Renshou, Meishan	KY283887	*F. graminearum*
37a*	wheat spike	Dongpo, Meishan	KY283888	*F. asiaticum*
39a*	wheat spike	Wenjiang, Chengdu	KY283889	*F. asiaticum*
42a*	wheat spike	Lezhi, Ziyang	KY283890	*F. asiaticum*
43a	wheat spike	Dujiangyan, Chengdu	KY283891	*F. asiaticum*

44a*	wheat spike	Dujiangyan, Chengdu	KY283892	*F. graminearum*
45a	wheat spike	Qionglai, Chengdu	KY283893	*F. asiaticum*
46a*	wheat spike	Dujiangyan, Chengdu	KY283894	*F. graminearum*
47a*	wheat spike	Lezhi, Ziyang	KY283895	*F. asiaticum*
48a*	wheat spike	Dujiangyan, Chengdu	KY283896	*F. asiaticum*
49a*	wheat spike	Dujiangyan, Chengdu	KY283897	*F. asiaticum*
50a	wheat spike	Zizhong, Neijiang	KY283898	*F. asiaticum*
51a	wheat spike	Longchang, Neijiang	KY283899	*F. asiaticum*
52a	wheat spike	Jianyang, Ziyang	KY283900	*F. meridionale*
53a	wheat spike	Jianyang, Ziyang	KY283901	*F. asiaticum*
54a	wheat spike	Jianyang, Ziyang	KY283902	*F. meridionale*
55a	wheat spike	Lezhi, Ziyang	KY283903	*F. asiaticum*
56a*	wheat spike	Gaoxian, Yibin	KY283904	*F. asiaticum*
57a*	wheat spike	Gaoxian, Yibin	KY283905	*F. asiaticum*
58a*	wheat spike	Xuanhan, Dazhou	KY283906	*F. asiaticum*
59a*	wheat spike	Dazhou, Dazhou	KY283907	*F. asiaticum*
60a	wheat spike	Dazhou, Dazhou	KY283908	*F. asiaticum*
61a*	wheat spike	Dujiangyan, Chengdu	KY283909	*F. asiaticum*
62a	wheat spike	Luojiang, Deyang	KY283910	*F. asiaticum*
63a	wheat spike	Luojiang, Deyang	KY283911	*F. asiaticum*
64a	wheat spike	Chuanshan, Suining	KY283912	*F. asiaticum*
65a	wheat spike	Jiangyou, Mianyang	KY283913	*F. asiaticum*
66a	wheat spike	Anzhou, Mianyang	KY283914	*F. asiaticum*
67a	wheat spike	Jiajiang, Leshan	KY283915	*F. asiaticum*
68a	wheat spike	Jiangyou, Mianyang	KY283916	*F. asiaticum*
69a*	wheat spike	Jiangyou, Mianyang	KY283917	*F. asiaticum*
70a*	wheat spike	Jiangyou, Mianyang	KY283918	*F. asiaticum*
71a	wheat spike	Jiangyou, Mianyang	KY283919	*F. asiaticum*
72a	wheat spike	Jiajiang, Leshan	KY283920	*F. asiaticum*
73a*	wheat spike	Jiajiang, Leshan	KY283921	*F. asiaticum*
74a*	wheat spike	Jiangyou, Mianyang	KY283922	*F. asiaticum*
75a*	wheat spike	Guanghan, Deyang	KY283923	*F. asiaticum*
76a*	wheat spike	Anzhou, Mianyang	KY283924	*F. asiaticum*
77a*	wheat spike	Jiajiang, Leshan	KY283925	*F. asiaticum*
78a*	wheat spike	Longchang, Neijiang	KY283926	*F. asiaticum*
79a	wheat spike	Jiajiang, Leshan	KY283927	*F. asiaticum*
80a*	wheat spike	Hejiang, Luzhou	KY283928	*F. asiaticum*
82a*	wheat spike	Luxian, Luzhou	KY283929	*F. asiaticum*
83a*	wheat spike	Linshui, Guang’an	KY283930	*F. asiaticum*
84a	wheat spike	Xichong, Nanchong	KY283931	*F. graminearum*
85a*	wheat spike	Luxian, Luzhou	KY283932	*F. asiaticum*
86a*	wheat spike	Luxian, Luzhou	KY283933	*F. asiaticum*
87a	wheat spike	Tianquan, Ya’an	KY283934	*F. asiaticum*

88a*	wheat spike	Gaoxian,Yibin	KY283935	*F. asiaticum*
89a*	wheat spike	Tianquan, Ya'an	KY283936	*F. asiaticum*
90a*	wheat spike	Cangxi, Guangyuan	KY283937	*F. asiaticum*
91a*	wheat spike	Cangxi, Guangyuan	KY283938	*F. asiaticum*
92a*	wheat spike	Guanghan, Deyang	KY283939	*F. asiaticum*
93a	wheat spike	Shifang, Deyang	KY283940	*F. asiaticum*
94a	wheat spike	Anzhou, Mianyang	KY283941	*F. asiaticum*
95a	wheat spike	Cangxi, Guangyuan	KY283942	*F. asiaticum*
96a*	wheat spike	Shifang, Deyang	KY283943	*F. asiaticum*
97a*	wheat spike	Cangxi, Guangyuan	KY283944	*F. asiaticum*
98a*	wheat spike	Cangxi, Guangyuan	KY283945	*F. asiaticum*
99a	wheat spike	Zitong, Mianyang	KY283946	*F. graminearum*
101a	wheat spike	Tongjiang, Bazhong	KY283947	*F. asiaticum*
102a	wheat spike	Enyang, Bazhong	KY283948	*F. asiaticum*
103a*	wheat spike	Linshui, Guang'an	KY283949	*F. asiaticum*
104a	wheat spike	Enyang, Bazhong	KY283950	*F. asiaticum*
105a	wheat spike	Tongjiang, Bazhong	KY283951	*F. asiaticum*
1S*	wheat spike	Xindu, Chengdu	n/a	*F. graminearum*
2S*	wheat spike	Xindu, Chengdu	n/a	*F. graminearum*
3S*	wheat spike	Pidu, Chengdu	n/a	*F. graminearum*
4S*	wheat spike	Shuangliu, Chengdu	n/a	*F. graminearum*
5S*	wheat spike	Qingbaijiang, Chengdu	n/a	*F. graminearum*
6S*	wheat spike	Dujiangyan, Chengdu	n/a	*F. graminearum*
7S*	wheat spike	Shuangliu, Chengdu	n/a	*F. graminearum*
8S*	wheat spike	Xindu, Chengdu	n/a	*F. graminearum*
9S*	wheat spike	Shuangliu, Chengdu	n/a	*F. graminearum*
10S*	wheat spike	Dujiangyan, Chengdu	n/a	*F. graminearum*
11S*	wheat spike	Wenjiang, Chengdu	n/a	*F. asiaticum*
12S*	wheat spike	Jintang, Chengdu	n/a	*F. asiaticum*
13S*	wheat spike	Xinjin, Chengdu	n/a	*F. asiaticum*
14S*	wheat spike	Chongzhou, Chengdu	n/a	*F. asiaticum*
15S*	wheat spike	Yanting, Mianyang	n/a	*F. graminearum*
16S*	wheat spike	Dujiangyan, Chengdu	n/a	*F. graminearum*
17S*	wheat spike	Bazhou, Bazhong	n/a	*F. asiaticum*
18S*	wheat spike	Jiajiang, Leshan	n/a	*F. asiaticum*
19S*	wheat spike	Jiajiang, Leshan	n/a	*F. asiaticum*
20S*	wheat spike	Jiajiang, Leshan	n/a	*F. asiaticum*
21S*	wheat spike	Tianquan, Ya'an	n/a	*F. graminearum*
22S*	wheat spike	Tianquan, Ya'an	n/a	*F. graminearum*
23S*	wheat spike	Tianquan, Ya'an	n/a	*F. graminearum*
24S*	wheat spike	Tianquan, Ya'an	n/a	*F. graminearum*
25S*	wheat spike	Tianquan, Ya'an	n/a	*F. graminearum*
26S*	wheat spike	Tianquan, Ya'an	n/a	*F. asiaticum*

27S*	wheat spike	Tianquan, Ya'an	n/a	*F. asiaticum*
28S*	wheat spike	Tianquan, Ya'an	n/a	*F. asiaticum*
29S*	wheat spike	Tianquan, Ya'an	n/a	*F. asiaticum*
30S*	wheat spike	Lushan, Ya'an	n/a	*F. asiaticum*
31S*	wheat spike	Tianquan, Ya'an	n/a	*F. asiaticum*
32S*	wheat spike	Tianquan, Ya'an	n/a	*F. asiaticum*
33S*	wheat spike	Tianquan, Ya'an	n/a	*F. asiaticum*
34S*	wheat spike	Tianquan, Ya'an	n/a	*F. asiaticum*
35S*	wheat spike	Nanbu,Nanchong	n/a	*F. asiaticum*
36S*	wheat spike	Xichong, Nanchong	n/a	*F. asiaticum*
37S*	wheat spike	Xichong, Nanchong	n/a	*F. asiaticum*
38S*	wheat spike	Nanbu,Nanchong	n/a	*F. asiaticum*
39S*	wheat spike	Gaoping,Nanchong	n/a	*F. asiaticum*
40S*	wheat spike	Xichong, Nanchong	n/a	*F. asiaticum*
41S*	wheat spike	Chuanshan, Suining	n/a	*F. graminearum*
42S*	wheat spike	Anzhou, Mianyang	n/a	*F. asiaticum*
43S*	wheat spike	Anzhou, Mianyang	n/a	*F. asiaticum*
44S*	wheat spike	Anzhou, Mianyang	n/a	*F. asiaticum*
45S*	wheat spike	Guanghan, Deyang	n/a	*F. graminearum*
46S*	wheat spike	Guanghan, Deyang	n/a	*F. graminearum*
47S*	wheat spike	Shifang, Deyang	n/a	*F. asiaticum*
48S*	wheat spike	Shifang, Deyang	n/a	*F. asiaticum*
49S*	wheat spike	Shifang, Deyang	n/a	*F. asiaticum*
50S*	wheat spike	Luojiang, Deyang	n/a	*F. asiaticum*
51S*	wheat spike	Luojiang, Deyang	n/a	*F. asiaticum*
52S*	wheat spike	Luojiang, Deyang	n/a	*F. asiaticum*
53S*	wheat spike	Lezhi, Ziyang	n/a	*F. graminearum*
54S*	wheat spike	Lezhi, Ziyang	n/a	*F. graminearum*
55S*	wheat spike	Yanjiang, Ziyang	n/a	*F. asiaticum*
56S*	wheat spike	Jianyang, Ziyang	n/a	*F. asiaticum*
57S*	wheat spike	Longchang, Neijiang	n/a	*F. asiaticum*
58S*	wheat spike	Zizhong, Neijiang	n/a	*F. asiaticum*
59S*	wheat spike	Zizhong, Neijiang	n/a	*F. asiaticum*
60S*	wheat spike	Longchang, Neijiang	n/a	*F. asiaticum*
61S*	wheat spike	Tongjiang, Bazhong	n/a	*F. asiaticum*
62S*	wheat spike	Bazhou, Bazhong	n/a	*F. asiaticum*
63S*	wheat spike	Bazhou, Bazhong	n/a	*F. asiaticum*
64S*	wheat spike	Enyang, Bazhong	n/a	*F. asiaticum*
65S*	wheat spike	Enyang, Bazhong	n/a	*F. asiaticum*
66S*	wheat spike	Daying, Suining	n/a	*F. graminearum*
67S*	wheat spike	Daying, Suining	n/a	*F. graminearum*
68S*	wheat spike	Chuanshan, Suining	n/a	*F. asiaticum*
69S*	wheat spike	Chuanshan, Suining	n/a	*F. asiaticum*

70S*	wheat spike	Daying, Suining	n/a	*F. asiaticum*
71S*	wheat spike	Quxian, Dazhou	n/a	*F. asiaticum*
72S*	wheat spike	Quxian, Dazhou	n/a	*F. asiaticum*
73S*	wheat spike	Luxian, Luzhou	n/a	*F. asiaticum*
74S*	wheat spike	Luxian, Luzhou	n/a	*F. asiaticum*
75S*	wheat spike	Luxian, Luzhou	n/a	*F. asiaticum*
76S*	wheat spike	Linshui, Guang'an	n/a	*F. asiaticum*
77S*	wheat spike	Daying, Suining	n/a	*F. graminearum*
HS1	maize stubble	Santai, Mianyang	KY435727	*F. asiaticum*
HS2	maize stubble	Santai, Mianyang	KY435728	*F. asiaticum*
HS3	maize stubble	Zhongjiang, Deyang	KY435729	*F. equiseti*
HS4	maize stubble	Chongzhou, Chengdu	KY435730	*F. asiaticum*
HS9	maize stubble	Santai, Mianyang	KY435731	*F. meridionale*
HS10	maize stubble	Qionglai, Chengdu	KY435732	*F. meridionale*
HS11	maize stubble	Santai, Mianyang	KY435733	*F. proliferatum*
HS13	maize stubble	Qionglai, Chengdu	KY435734	*F. asiaticum*
HS14	maize stubble	Zhongjiang, Deyang	KY435735	*F. graminearum*
HS15	maize stubble	Qionglai, Chengdu	KY435736	*F. asiaticum*
HS17	maize stubble	Dayi, Chengdu	KY435737	*F. temperatum*
HS18	maize stubble	Dayi, Chengdu	KY435738	*F. temperatum*
HS19	maize stubble	Dayi, Chengdu	MH684481	*F. asiaticum*
HS20	maize stubble	Zhongjiang, Deyang	KY435739	*F. graminearum*
HS21	maize stubble	Zhongjiang, Deyang	MH684485	*F. temperatum*
HS22	maize stubble	Zhongjiang, Deyang	KY435740	*F. graminearum*
HS23	maize stubble	Zhongjiang, Deyang	MH684482	*F. asiaticum*
HS24	maize stubble	Qionglai, Chengdu	KY435741	*F. graminearum*
HS25	maize stubble	Qionglai, Chengdu	KY435742	*F. graminearum*
HS26	maize stubble	Qionglai, Chengdu	KY435743	*F. meridionale*
HS27	maize stubble	Qionglai, Chengdu	KY435744	*F. asiaticum*
HS31	maize stubble	Yucheng, Ya'an	KY435745	*F. graminearum*
HS32	maize stubble	Pidu, Chengdu	KY435746	*F. graminearum*
HS33	maize stubble	Pidu, Chengdu	MH684483	*F. asiaticum*
HS34	maize stubble	Yucheng, Ya'an	KY435747	*F. graminearum*
HS36	maize stubble	Qionglai, Chengdu	KY435748	*F. meridionale*
HS37	maize stubble	Qionglai, Chengdu	KY435749	*F. meridionale*
HS38	maize stubble	Dayi, Chengdu	KY435750	*F. graminearum*
HS39	maize stubble	Dayi, Chengdu	MH684484	*F. asiaticum*
HS40	maize stubble	Yanting, Mianyang	KY435751	*F. proliferatum*
HS46	maize stubble	Pixian, Chengdu	KY435752	*F. graminearum*
HS47	maize stubble	Zhongjiang, Deyang	KY435753	*F. equiseti*
1b	wheat straw	Chongzhou, Chengdu	KY466689	*F. graminearum*
2b	wheat straw	Chongzhou, Chengdu	KY466690	*F. graminearum*
3b	wheat straw	Chongzhou, Chengdu	KY466691	*F. graminearum*

4b	wheat straw	Chongzhou, Chengdu	KY466692	*F. graminearum*
5b	wheat straw	Pidu, Chengdu	KY466693	*F. graminearum*
6b	wheat straw	Chongzhou, Chengdu	KY466694	*F. graminearum*
7b	wheat straw	Chongzhou, Chengdu	KY466695	*F. graminearum*
10b	wheat straw	Pidu, Chengdu	KY466696	*F. graminearum*
11b	wheat straw	Chongzhou, Chengdu	KY466697	*F. graminearum*
12b	wheat straw	Chongzhou, Chengdu	KY466698	*F. graminearum*
13b	wheat straw	Chongzhou, Chengdu	KY466699	*F. graminearum*
15b	wheat straw	Pidu, Chengdu	KY466700	*F. graminearum*
16b	wheat straw	Pidu, Chengdu	KY466701	*F. graminearum*
18b	wheat straw	Chongzhou, Chengdu	KY466702	*F. graminearum*
20b	wheat straw	Chongzhou, Chengdu	KY466703	*F. graminearum*
21b	wheat straw	Chongzhou, Chengdu	KY466704	*F. graminearum*
22b	wheat straw	Chongzhou, Chengdu	KY466705	*F. graminearum*
23b	wheat straw	Yucheng, Ya'an	KY466706	*F. asiaticum*
25b	wheat straw	Chongzhou, Chengdu	KY466707	*F. graminearum*
26b	wheat straw	Pidu, Chengdu	KY466708	*F. graminearum*
27b	wheat straw	Pidu, Chengdu	KY466709	*F. graminearum*
29b	wheat straw	Chongzhou, Chengdu	KY466710	*F. graminearum*
31b	wheat straw	Chongzhou, Chengdu	KY466711	*F. graminearum*
32b	wheat straw	Yucheng, Ya'an	KY466712	*F. meridionale*
33b	wheat straw	Chongzhou, Chengdu	KY466713	*F. graminearum*
34b	wheat straw	Chongzhou, Chengdu	KY466714	*F. graminearum*
35b	wheat straw	Chongzhou, Chengdu	KY466715	*F. equiseti*
36b	wheat straw	Chongzhou, Chengdu	KY466716	*F. asiaticum*
37b	wheat straw	Yucheng, Ya'an	KY466717	*F. meridionale*
38b	wheat straw	Yucheng, Ya'an	KY466718	*F. meridionale*
39b	wheat straw	Yucheng, Ya'an	KY466719	*F. meridionale*
40b	wheat straw	Chongzhou, Chengdu	KY466720	*F. graminearum*
41b	wheat straw	Chongzhou, Chengdu	KY466721	*F. graminearum*
42b	wheat straw	Chongzhou, Chengdu	KY466722	*F. asiaticum*
43b	wheat straw	Chongzhou, Chengdu	KY466723	*F. graminearum*
44b	wheat straw	Chongzhou, Chengdu	KY466724	*F. graminearum*
45b	wheat straw	Chongzhou, Chengdu	KY466725	*F. graminearum*
46b	wheat straw	Chongzhou, Chengdu	KY466726	*F. graminearum*
47b	wheat straw	Chongzhou, Chengdu	KY466727	*F. graminearum*
48b	wheat straw	Chongzhou, Chengdu	KY466728	*F. graminearum*
49b	wheat straw	Chongzhou, Chengdu	KY466729	*F. graminearum*
50b	wheat straw	Chongzhou, Chengdu	KY466730	*F. asiaticum*
51b	wheat straw	Yucheng, Ya'an	KY466731	*F. meridionale*
52b	wheat straw	Yucheng, Ya'an	KY466732	*F. meridionale*
53b	wheat straw	Yucheng, Ya'an	KY466733	*F. meridionale*
54b	wheat straw	Chongzhou, Chengdu	KY466734	*F. asiaticum*

55b	wheat straw	Yucheng, Ya’an	KY466735	*F. meridionale*
56b	wheat straw	Dayi, Chengdu	KY466736	*F. asiaticum*
57b	wheat straw	Dayi, Chengdu	KY466737	*F. asiaticum*
58b	wheat straw	Dayi, Chengdu	KY466738	*F. graminearum*
59b	wheat straw	Dayi, Chengdu	KY466739	*F. asiaticum*
60b	wheat straw	Dayi, Chengdu	KY466740	*F. asiaticum*
61b	wheat straw	Dayi, Chengdu	KY466741	*F. asiaticum*
62b	wheat straw	Chongzhou, Chengdu	KY466742	*F. graminearum*
63b	wheat straw	Zhongjiang, Deyang	KY466743	*F. asiaticum*
64b	wheat straw	Zhongjiang, Deyang	KY466744	*F. asiaticum*
65b	wheat straw	Zhongjiang, Deyang	KY466745	*F. graminearum*
66b	wheat straw	Renshou, Meishan	KY466746	*F. asiaticum*
67b	wheat straw	Zhongjiang, Deyang	KY466747	*F. asiaticum*
68b	wheat straw	Zhongjiang, Deyang	KY466748	*F. graminearum*
69b	wheat straw	Zhongjiang, Deyang	KY466749	*F. asiaticum*
71b	wheat straw	Zhongjiang, Deyang	KY466750	*F. graminearum*
72b	wheat straw	Renshou, Meishan	KY466751	*F. asiaticum*
79b	wheat straw	Zhongjiang, Deyang	KY466752	*F. asiaticum*
82b	wheat straw	Dayi, Chengdu	KY466753	*F. graminearum*
83b	wheat straw	Renshou, Meishan	KY466754	*F. asiaticum*
84b	wheat straw	Qionglai, Chengdu	KY466755	*F. asiaticum*
85b	wheat straw	Qionglai, Chengdu	KY466756	*F. asiaticum*
86b	wheat straw	Qionglai, Chengdu	KY466757	*F. asiaticum*
87b	wheat straw	Renshou, Meishan	KY466758	*F. asiaticum*
88b	wheat straw	Pidu, Chengdu	KY466759	*F. asiaticum*
89b	wheat straw	Pidu, Chengdu	KY466760	*F. asiaticum*
90b	wheat straw	Pidu, Chengdu	KY466761	*F. asiaticum*
91b	wheat straw	Zhongjiang, Deyang	KY466762	*F. asiaticum*
92b	wheat straw	Pidu, Chengdu	KY466763	*F. asiaticum*
94b	wheat straw	Santai, Mianyang	KY466764	*F. asiaticum*
95b	wheat straw	Santai, Mianyang	KY466765	*F. asiaticum*
96b	wheat straw	Santai, Mianyang	KY466766	*F. asiaticum*
97b	wheat straw	Qionglai, Chengdu	KY466767	*F. asiaticum*
98b	wheat straw	Santai, Mianyang	KY466768	*F. asiaticum*
99b	wheat straw	Santai, Mianyang	KY466769	*F. asiaticum*
101b	wheat straw	Qionglai, Chengdu	KY466770	*F. asiaticum*
102b	wheat straw	Qionglai, Chengdu	KY466771	*F. asiaticum*
106b	wheat straw	Dayi, Chengdu	KY466772	*F. meridionale*
107b	wheat straw	Dayi, Chengdu	KY466773	*F. meridionale*
108b	wheat straw	Dayi, Chengdu	KY466774	*F. meridionale*
111b	wheat straw	Dayi, Chengdu	KY466775	*F. asiaticum*
114b	wheat straw	Zhongjiang, Deyang	KY466776	*F. asiaticum*
115b	wheat straw	Dayi, Chengdu	KY466777	*F. asiaticum*

116b	wheat straw	Renshou, Meishan	KY466778	*F. asiaticum*
119b	wheat straw	Dayi, Chengdu	KY466779	*F. meridionale*
120b	wheat straw	Dayi, Chengdu	KY466780	*F. graminearum*
121b	wheat straw	Dayi, Chengdu	KY466781	*F. asiaticum*
122b	wheat straw	Qionglai, Chengdu	KY466782	*F. asiaticum*
123b	wheat straw	Dayi, Chengdu	KY466783	*F. meridionale*
124b	wheat straw	Dayi, Chengdu	KY466784	*F. asiaticum*
125b	wheat straw	Dayi, Chengdu	KY466785	*F. asiaticum*
127b	wheat straw	Renshou, Meishan	KY466786	*F. asiaticum*
128b	wheat straw	Dayi, Chengdu	KY466787	*F. asiaticum*
129b	wheat straw	Qionglai, Chengdu	KY466788	*F. proliferatum*
130b	wheat straw	Qionglai, Chengdu	KY466789	*F. proliferatum*
131b	wheat straw	Dayi, Chengdu	KY466790	*F. asiaticum*
132b	wheat straw	Renshou, Meishan	KY466791	*F. proliferatum*
133b	wheat straw	Renshou, Meishan	KY466792	*F. graminearum*

Notes: *TEF1α*, translation elongation factor 1α; n/a = not available. * Isolates were analyzed using the primer pair Fg16F/Fg16R.
